# Analysis of microbial sequences in plasma cell-free DNA for early-onset breast cancer patients and healthy females

**DOI:** 10.1186/s12920-018-0329-y

**Published:** 2018-02-13

**Authors:** Yu-Feng Huang, Yen-Ju Chen, Tan-Chi Fan, Nai-Chuan Chang, Yi-Jie Chen, Mohit K. Midha, Tzu-Han Chen, Hsiao-Hsiang Yang, Yu-Tai Wang, Alice L. Yu, Kuo-Ping Chiu

**Affiliations:** 1 Genomics Research Center, Academia Sinica, 128 Academia Road, Section 2, Nankang District, Taipei, 115 Taiwan; 2 Institute of Stem Cell and Translational Cancer Research, Chang Gung Memorial Hospital at Linkou, Chang Gung University, No. 5, Fu-Shin St., Kuei Shang, Taoyuan, 333 Taiwan; 3 Institute of Biochemistry and Molecular Biology, National Yang-Ming University, Taipei, Taiwan; 4 National Center for High-Performance Computing, Hsinchu Science Park, Hsinchu, Taiwan; 5 Department of Pediatrics, University of California in San Diego, San Diego, CA USA; 6 Department of Life Sciences, College of Life Sciences, National Taiwan University, Taipei, Taiwan

**Keywords:** Cell-free circulating DNA (cfDNA), Microbial cfDNA reads per million quality PE reads (MCRPM), Microbial cfDNA

## Abstract

**Background:**

Cell-free circulating DNA (cfDNA) is becoming a useful biopsy for noninvasive diagnosis of diseases. Microbial sequences in plasma cfDNA may provide important information to improve prognosis and treatment. We have developed a stringent method to identify microbial species via microbial cfDNA in the blood plasma of early-onset breast cancer (EOBC) patients and healthy females. Empirically, microbe-originated sequence reads were identified by mapping non-human PE reads in cfDNA libraries to microbial databases. Those mapped concordantly to unique microbial species were assembled into contigs, which were subsequently aligned to the same databases. Microbial species uniquely aligned were identified and compared across all individuals on MCRPM (Microbial CfDNA Reads Per Million quality PE reads) basis.

**Results:**

The predominant microbial cfDNAs in all plasma samples examined are originated from bacteria and these bacteria were limited to only a few genera. Among those, *Acinetobacter johnsonii* XBB1 and low levels of Mycobacterium spp. were commonly found in all healthy females, but also present in an EOBC patient. Compared to those in healthy counterparts, bacterial species in EOBC patients are more diverse and more likely to present at high levels. Among these three EOBC patients tested, a patient who has record high titer (2,724 MCRPM) of *Pseudomonas mendocina* together with 8.82 MCRPM of *Pannonibacter phragmitetus* has passed away; another patient infected by multiple Sphingomonas species remains alive; while the third patient who has similar microbial species (*Acinetobacter johnsonii* XBB1) commonly seen in normal controls is having a normal life.

**Conclusions:**

Our preliminary data on the profiles of microbial cfDNA sequences suggested that it may have some prognostic value in cancer patients. Validation in larger number of patients is warranted.

## Background

It is recently established that human blood contains cell-free circulating DNA (cfDNA) resulted from apoptosis and necrosis of normal and diseased human cells or inhabited microbes [1]. Microbial genomic DNA can be detected in plasma [2, 3], and the level of bacterial sequences in cfDNA was found to be higher for diseased and immunocompromised individuals than healthy counterparts [4]. A number of studies have also indicated that a low level of pleomorphic bacteria may inhabit normal blood circulation [5–7], and bacteria were also found in tumor tissues [8]. Data have also shown that these microbes may regulate a broad range of host physiological activities including metabolism, inflammation, immunity and hematopoiesis, which may in turn influence tumorigenesis and/or cancer development [9]. These data suggest that microbiota may play a key role to orchestrate cancer therapy and many more cancer-associated microbes need to be identified.

It was estimated that 15–20% of human cancers were caused by viral infections [10, 11]. The best-known and best-studied carcinogenic microbes are viruses. These include hepatitis B (HBV) and C (HCV) viruses, human papillomavirus (HPV), human T-cell leukemia virus and Epstein-Barr virus (EBV) [12–16]. The discoveries of viral roles in tumorigenesis were facilitated by the fact that viruses are absolute parasites and that viral genomes are generally smaller and with fewer genes compared to bacterial and fungal genomes. Although reports have shown the presence of microbes in human breast cancer tissues and lateral gene transfer of microbial genetic sequences into the human genome, especially in cancer tissues [17, 18]. Bacteria and fungi have complex genomes and their interactions with hosts are more intricate than that of viruses, making it more difficult to justify their roles in tumorigenesis [18].

With the robust speed and yield of next-generation sequencing (NGS) [19], in combination with the noninvasiveness and easy accessibility of liquid biopsies and the availability of microbial genome databases, NGS-based cfDNA sequencing is a powerful approach for studying microbiota in body fluids. Recently, Grumaz and colleagues have demonstrated that NGS-coupled microbial cfDNA analysis is strongly correlated with the results of blood cultures [3]. Besides the Gram-negative bacteria which exactly matched the results from corresponding blood culturing, they also found microbial species showing false negative in blood culture, indicating the superiority of the approach. Their work represents a proof-of-concept pioneer work for direct detection of bacterial species using plasma cfDNA.

Here, we present another NGS-based approach which is able to identify microbes of a broad range. Our method relies on a stringent mapping/alignment procedure for specific association of microbial cfDNA sequences with potential pathogens and rare microbial species which may present in cancer patients and/or healthy individuals. Empirically, paired-end (PE) reads unmappable to hg19 were collected and mapped to microbial databases composed of bacterial, fungal and viral/phage genomes. Those mapped concordantly to unique microbial species were assembled into contigs, which were subsequently used to BLAST the same microbial databases. The microbial species aligned uniquely by contigs were considered as the real hits. Microbes associated with each person were portrayed with their relative microbial cfDNA levels on a MCRPM (Microbial CfDNA Reads Per Million quality PE reads) basis.

In this study, we identified microbial species in both healthy and diseased females. One of the most striking features is that the microbial inhabitants in breast cancer patients are not only much more diverse than that of normal females, but also can be tightly correlated to the health conditions of the patients. Taken together, this investigation indicates that NGS-based microbial cfDNA sequencing has a strong potential to improve disease diagnosis and treatment.

## Results and discussion

### Microbial databases

Genomic sequences of bacteria, fungi and viruses were downloaded from NCBI (April 6, 2017). Plasmids were subsequently removed. The database contain 9,336, 913 and 9,050 genomes of bacteria, fungi and viruses/phages, respectively (Table 1).

**Table 1:** Microbial databases employed in the study

	Bacteria	Fungi	Viruses
#Contigs	39,434	20	0
#Scaffold	36,076	170	3
#Chromosome	978	38	22
#Complete genome	6,711	7	7,175
#Subtotal	83,199	235	7,200
#Species	7,689	45	7,197
#Sequences	15,849	913	9,050
Total #, after plasmid sequences excluded	9,336	913	9,050

RefSeq genome: April 6, 2017

### Library statistics

Five plasma DNA libraries including BBC (normal), EJC (normal), BC0145 (EOBC), BC0190 (EOBC) and CGBC025 (EOBC) were sequenced by Illumina HiSeq X Ten to produce at least 384 million PE reads per library (eq. ~ 38-fold coverage) (Table 2). Mapping against human genome assembly hg19 resulted in mappable ratios of 94.62%, 96.79%, 92.13%, 95.99% and 95.16% (eq. to 5.38%, 3.21%, 7.87%, 4.01% and 4.84% of unmappable ratios) for BBC, EJC, BC0145, BC0190 and CGBC025 libraries, respectively.

**Table 2:** Library statistics

Library	BBC (normal)	EJC (normal)	BC0145 (EOBC)	BC0190 (EOBC)	CGBC025 (EOBC)
Raw PE reads	384,623,309	420,790,943	392,322,204	434,928,103	628,712,403
Quality PE reads	371,837,085	388,551,037	375,255,962	426,187,686	579,192,276
Normalization factor	372	389	375	426	579
hg19-mapped reads (%)	351,841,885 (94.62%)	376,097,300 (96.79%)	345,714,693 (92.13%)	409,083,110 (95.99%)	551,181,421 (95.16%)
hg19-unmappable PE reads (%)	19,995,200 (5.38%)	12,453,737 (3.21%)	29,541,269 (7.87%)	17,104,576 (4.01%)	28,010,855 (4.84%)

### Most of the microbial cfDNA sequences were originated from bacteria, instead of fungi or viruses/phages

Non-human PE reads (i.e., reads unmappable to hg19) were mapped against the microbial databases. 0.08% - 4.85% (per library) were found to be originated from bacteria, 0.00% - 0.01% from fungi, and 0.00% - 0.16% from viruses/phages (Table 3, upper part). PE reads uniquely and concordantly mapped to single species were then used to build contigs. When contigs were aligned to the same microbial databases, numbers of contigs aligned to bacteria were also significantly higher than that aligned to either fungi or viruses/phages for all libraries (Table 3, lower part).

**Table 3:** Statistics of contigs and alignment

		BBC	EJC	BC0145	BC0190	CGBC025
hg19-unmapped PE reads		19,995,200	12,453,737	29,541,269	17,104,576	28,010,855
Bacteria	Mapped PE reads (%)	15,504 (0.08%)	31,653 (0.25%)	1,432,423 (4.85%)	50,310 (0.29%)	45,858 (0.16%)
Fungi	Mapped PE reads (%)	451 (0.00%)	456 (0.00%)	590 (0.00%)	1,153 (0.01%)	996 (0.00%)
Viruses/Phages	Mapped PE reads (%)	1,367 (0.01%)	677 (0.01%)	1,528 (0.01%)	26,749 (0.16%)	1,267 (0.00%)
Bacteria	#Contigs	894	1,495	7,971	2,609	2,588
	Max contig length	900	2,287	16,248	2,035	3,911
	Min contig length	64	64	64	64	64
	Median contig length	211	227	231	214	220
	N50	215	266	1,616	219	237
	#contigs w/ size ≥250 bp (%)	80 (9.0%)	558 (37.3%)	3,602 (45.2%)	333 (12.8%)	748 (28.9%)
	#aligned contigs (also see Table 4)	54	381	2,456	225	451
Fungi	#contigs	44	38	215	92	78
	Max contig length	315	235	352	427	262
	Min contig length	65	64	64	64	64
	Median contig length	71	84	71	84	73.5
	N50	99	192	78	192	127
	#contigs w/ size ≥250 bp (%)	2 (4.6%)	0 (0.0%)	3 (1.4%)	3 (3.3%)	1 (1.3%)
	#aligned contigs	2	0	0	2	1
Viruses/ phages	#Contigs	75	62	287	155	72
	Max contig length	655	546	336	695	274
	Min contig length	64	64	64	64	64
	Median contig length	68	84	70	73	74
	N50	194	210	85	189	126
	#contigs w/ size ≥250 bp (%)	9 (12.0%)	7 (11.3%)	10 (3.5%)	13 (8.4%)	1 (1.4%)
	#aligned contigs	7	5	2	10	0

### Most bacterial cfDNA in healthy females are derived from Acinetobacter spp., while high titers of bacterial cfDNA in EOBC patients are derived from Pseudomonas or Sphingomonas spp.

To compare the levels (or titers) of microbial cfDNA fragments between individuals, the constituent sequence reads of all contigs were summed up for each microbe and normalized on per-million basis (see Normalization in Methods section). This strategy is able to reflect the relative levels for the cfDNA fragment compositions in the blood, because each read was supposed to be resulted from a cfDNA fragment.

Alignment with contigs maximizes the accuracy of microbial species identification, while read-level normalization aims to achieve the best approximation of relative titers for microbial species identified. Microbial species mapped with single contigs were also recorded because the number of constituent reads may vary significantly and can be very high for long contigs. Notice that each read represents a cfDNA fragment either in the original plasma sample or produced by PCR amplification. To minimize the impact of nonspecific alignment, bacterial species with < 0.1 MCRMP were removed from the list.

Bacterial species identified are significantly different between healthy and diseased individuals (except BC0190) (Table 4). As indicated by non-parametric Mann-Whitney-Wilcoxon test, bacterial species found between normal and diseased individuals are different (*p*-value 0.020). Healthy females tend to harbor Acinetobacter species, especially *Acinetobacter johnsonii XBB1*, although the level may fluctuate (ranging between 0.27–6.00 MCRPM). Mycobacterium spp. and *Ralstonia mannitolilytica* were also present in healthy females (and BC0190 as well), but less significant. On the other hand, EOBC patients tend to harbor more diverse bacterial species, presumably influenced by secondary infection. The highest titer (2742.72 MCRPM) was found to be *Pseudomonas mendocina ymp* in patient BC0145, while the second highest titer (13.64 MCRPM) was *Sphingomonas melonis* in patient CGBC025. Patient BC0145, who was diagnosed with bilateral breast cancer and passed away a year after surgery in 2007 (see Plasma cfDNA preparation in Methods section), was also infected by other Pseudomonas spp. (e.g., *P. alcaliphila* of 2.84 MCRPM and *P. aeruginosa* of 0.89 MCRPM) and *Pannonibacter phragmitetus* (8.82 MCRPM). In contrast to patients BC0145 and CGBC025 but similar to normal females, patient BC0190 had *Acinetobacter johnsonii* XBB1 as the major bacterial inhabitant. She had surgery in February of 2007 and since then has been living a healthy life.

**Table 4:** Bacterial species identified

ID	Species	No. of contigs	Total aligned length	Total no. of associated PE reads	Total no. of associated SE reads	Sum of PE and SE reads	MCRPM
BBC ctl							(sum/372)
	*Acinetobacter johnsonii* XBB1	17	5,428	69	30	99	0.27
	*Bacillus cereus*	2	708	6	35	41	0.11
	*Mycobacterium sp.*	4	2,124	73	70	143	0.38
	*Ralstonia mannitolilytica*	4	1,227	31	12	43	0.12
EJC ctl							(sum/389)
	*Acinetobacter haemolyticus*	12	4,345	59	30	89	0.23
	***Acinetobacter johnsonii*** **XBB1**	231	99,403	1,719	616	2,335	**6.00**
	*Acinetobacter junii*	4	1,948	30	7	37	0.10
	*Acinetobacter lwoffii*	17	6,186	87	31	118	0.30
	*Acinetobacter schindleri*	4	1,770	43	16	59	0.15
	*Bacillus cereus*	4	1,314	31	51	82	0.21
	*Corynebacterium pseudotuberculosis*	2	588	2	48	50	0.13
	*Methylobacterium populi*	18	5,102	54	18	72	0.19
	*Mycobacterium abscessus*	4	2,238	53	14	67	0.17
	*Mycobacterium avium*	8	2,751	30	15	45	0.12
	*Mycobacterium sp.*	8	2,620	36	63	99	0.25
	*Mycoplasma mycoides*	1	276	17	28	45	0.12
	*Porphyrobacter sp.*	7	2,478	20	34	54	0.14
	*Ralstonia mannitolilytica*	8	2,899	37	40	77	0.20
	*Sphingomonas sp.*	11	3,533	27	28	55	0.14
BC 0145							(sum/375)
	***Pannonibacter phragmitetus***	714	230,779	1,999	1,309	3,308	**8.82**
	*Pseudomonas aeruginosa*	2	1,824	254	78	332	0.89
	***Pseudomonas alcaliphila***	6	5,093	896	170	1,066	**2.84**
	***Pseudomonas mendocina ymp***	1,675	2,678,493	918,733	109,787	1,028,520	**2,742.72**
	*Pseudomonas sp.*	3	1,195	41	36	77	0.21
	*Pseudomonas stutzeri*	3	1,717	220	21	241	0.64
	*Rhizobium sp.*	34	12,418	144	65	209	0.56
BC 0190							(sum/426)
	*Acinetobacter baumannii*	2	1,078	29	17	46	0.11
	***Acinetobacter johnsonii XBB1***	71	24,930	366	168	534	**1.25**
	*Bacillus cereus*	4	1,172	24	76	100	0.23
	*Methylobacterium populi*	38	10,970	123	72	195	0.46
	*Mycobacterium abscessus*	5	2,599	67	28	95	0.22
	*Mycobacterium sp.*	7	2,432	52	88	140	0.33
	*Porphyrobacter sp.*	6	2,195	38	31	69	0.16
	*Ralstonia mannitolilytica*	7	2,370	19	53	72	0.17
	*Sphingomonas sp.*	17	5,030	63	44	107	0.25
CGBC 025							(sum/579)
	*Acinetobacter baumannii*	24	6,818	118	48	166	0.29
	*Bradyrhizobium sp.*	7	2,059	41	16	57	0.10
	*Enterobacter cloacae*	8	3,140	108	33	141	0.24
	*Sphingobium japonicum*	8	5,088	262	43	305	0.53
	***Sphingomonas melonis***	336	157,346	5,397	2,503	7,900	**13.64**
	*Sphingomonas panacis*	4	1,261	36	30	66	0.11
	***Sphingomonas sp.***	51	21,410	720	271	991	**1.71**

Only microbial species with MCRPM ≥ 0.1 are listed. Those with MCRPM ≥ 1 are listed in bold.

*Ctl* control; MCRPM, microbial cfDNA per million quality PE reads; *sp. (same as spp.),* species with unspecified species name

*Acinetobacter johnsonii* (Gram-negative (G-)) is a ubiquitous bacterium commonly found in human and fish [20–22]. However, it is becoming an emerging threat to human health [23]. *A. johnsonii* XBB1 was first isolated from a hospital in Chengdu City, China. Both Mycobacteria and *Ralstonia mannitolilytica* are also ubiquitous in nature. *R. mannitolilytica* (G-), which was ever named as *Pseudomonas thomasii*, *Pseudmonas pickettii* or *Ralstonia pickettii*, is a soil bacterium. In 1989, this bacterium caused a nosocomial outbreak in Taiwan through sodium chloride contamination [24]. *Sphingomonas melonis* was first isolated from melon. Strain *Sphingomonas melonis* sp. nov. is becoming a common pathogen in melon fruits [25]. This species was detected in both healthy and diseased females and a high titer (13.64 MCRPM) was observed in patient CGBC025.

Most of the bacteria on the list (even Pseudomonas spp.) are known as opportunistic bacterial pathogens. For example, *P. aeruginosa* (G-) is a bacterium commonly found in skin of healthy people [26]. Both *R. mannitolilytica*, *P. aeruginosa* are innocuous bacteria, but can be acquired from hospital where antibiotics are commonly used. Through constant challenge by novel and multiple antibiotics, they might have become multidrug resistant through horizontal transfer.

### Fungal viral and phage species

When aligned to fungal and viral/phage databases, PE reads of all five libraries showed no significant hit for all individuals, either healthy or diseased. Thus, for all these categories, results are much less significant than that of bacterial species.

## Conclusions

As revealed by previous investigations, microbes may inhabit a number of human tissues which are technically inaccessible for direct analysis. Fortunately, their genomic sequences are released into blood circulation and are thus become available for consecutive experimentation for diagnostic or prognostic purposes. As such, NGS-based cfDNA sequencing has emerged to be one of the most suitable choices.

Here, we empirically demonstrate the feasibilities of using NGS-based cfDNA sequencing for direct detection of microbial species in human body. The feasibility is made possible by NGS, the noninvasiveness and easy accessibility of plasma cfDNA samples, and a stringent protocol for mapping and alignment.

The process is guarded by multiple layers of stringency. Firstly, it started only with PE reads which map concordantly to unique microbial genomes; both discordant hits and single-end hits were excluded in this step, although single-end matches may be included during contig assembly. Secondly, since microbes share significant degree of sequence similarity, contigs were built and used to align to the same microbial genome assemblies for better accuracy. Thirdly, contig-level alignment was reinforced with stringent parameters. Fourthly, only those microbial species “uniquely” aligned by contigs were considered as real hits. Moreover, microbial spp. with less than 0.1 MCRPM were excluded.

Although only five samples were analyzed, this study reveals a number of interesting phenomena: **1)** microbial cfDNA can also be detected in the blood plasma of healthy individuals; **2)** bacteria constitute the major microbial species in human body; **3)** these bacteria belong to only a limited number of genera and species, no matter for healthy or diseased individuals; **4)** most microbial species in healthy human bodies show only low levels of cfDNA; **5)** Acinetobacter (esp., *A. johnsonii* XBB1) cfDNA is ubiquitous for healthy individuals, but it may also present in EOBC patient with near normal heath conditions; **6)** on the other hand, Pseudomonas and Sphingomonas cfDNA can be found in the plasma of breast cancer patients with severe symptoms; **7)** microbial spp. in breast cancer patients tend to be more diverse compared to that in healthy counterparts.

Pseudomonas species are well known opportunistic bacteria commonly found on the surface of human body. It is thus not surprising to detect Pseudomonas in cancer patients with symptoms severe enough to compromise the immune system. On the other hand, the presence of *Sphingomonas melonis* in a cancer patient is somewhat surprising. In natural environment, this bacterium is known to cause brown spots on yellow Spanish melons. Its pathogenicity upon human was first described only 15 years ago [25]. Its quick spreading is a warning sign to human communities.

For cancer or other diseases resulted from viruses living constantly in human body (e.g., HPV and HSV which cause cervical cancer and herpes respectively), the presence of corresponding viral cfDNA sequences in plasma are expected. However, the viral cfDNA titer is expected to fluctuate with its proliferation status, with relatively higher titer during active stage and lower titer during dormant stage. For breast cancer, no clear correlation with viral pathogens has ever been unambiguously demonstrated. This partially explains why no significant viral cfDNA were detected among these EOBC patients.

Furthermore, the microbial species detected in EOBC patients may not be responsible for the tumorigenesis of the disease, because, as influenced by health conditions as well as environmental factors, the composition of microbial species in human body can be very dynamic and the cfDNA sequences of the causative agents may no longer present or may present only at an insignificant level when the disease is eventually established. However, our results indicate a correlation between disease status and the composition of microbial cfDNA in plasma, and suggest the feasibility of using NGS-based microbial cfDNA sequencing for personalized treatment of diseases.

Although this study allows us to extrapolate certain interesting phenomena from microbial cfDNA in plasma, the capacity of our approach is limited by sample size. We intend to survey large number of healthy and diseased individuals in the future, including patients with different types of cancer and/or different cancer stages. Besides cancer, it would also be interesting to expand the approach to other diseases, especially chronic diseases such as Type I and Type II diabetes. Through analysis of microbial cfDNA in plasma, we should be able to gain further insight into the role played by microbial inhabitants during the progression of a disease. Knowledge of this kind should be able to facilitate the effectiveness of personalized treatment of diseases.

## Methods

### Plasma cfDNA preparation

Detailed information about the EOBC patients: **1)** Patient BC0145 (ER-, PR+ and HER-2+) was diagnosed at age 39.5 to have infiltrating ductal carcinoma at grade III in both breasts, had surgery on August 22, 2006 and passed away on September 01, 2007 (about 1 year after surgery). **2)** Patient BC0190 (ER+, PR+ and HER-2+) was diagnosed at age 25.1 to have infiltrating ductal carcinoma at grade III, had her surgery on February 16, 2007 and was reported to remain healthy in April of 2017. **3)** Patient CGBC025 (ER+, PR+ and HER-2+) was diagnosed at age 34.8 to have invasive ductal carcinoma at grade II, had her surgery on August 28, 2015 and remains alive and healthy.

Blood samples were taken from EOBC patients BC0145, BC0190 and CGBC025 at age 39.5, 25.1 and 34.8, respectively, and normal blood samples were prepared from healthy females BBC and EJC, both less than 30 years old. All plasma cfDNA samples were isolated from blood using Quick-cfDNA Serum and Plasma Kit (Zymo Research, D4076) following manufacturer’s instructions.

### Preparation of microbial reference databases

Microbial reference databases were downloaded from NCBI reference genome database (ftp://ftp.ncbi.nlm.nih.gov/genomes/refseq/, April 6, 2017). For each category, only “Complete Genome” and “Chromosome” from assembly_summary.txt according guide line from NCBI web site (https://www.ncbi.nlm.nih.gov/genome/doc/ftpfaq/#allcomplete) were used in mapping/alignment. The final reference databases contain 9,336 bacterial sequences, 9,050 viral sequences, and 913 fungal sequences (Table 1).

### Sequencing and sequence processing

Sequences were determined by 150 × 150 PE sequencing (30×) using Illumina HiSeq X Ten (Macrogen Corp).

**Fig. 1:**
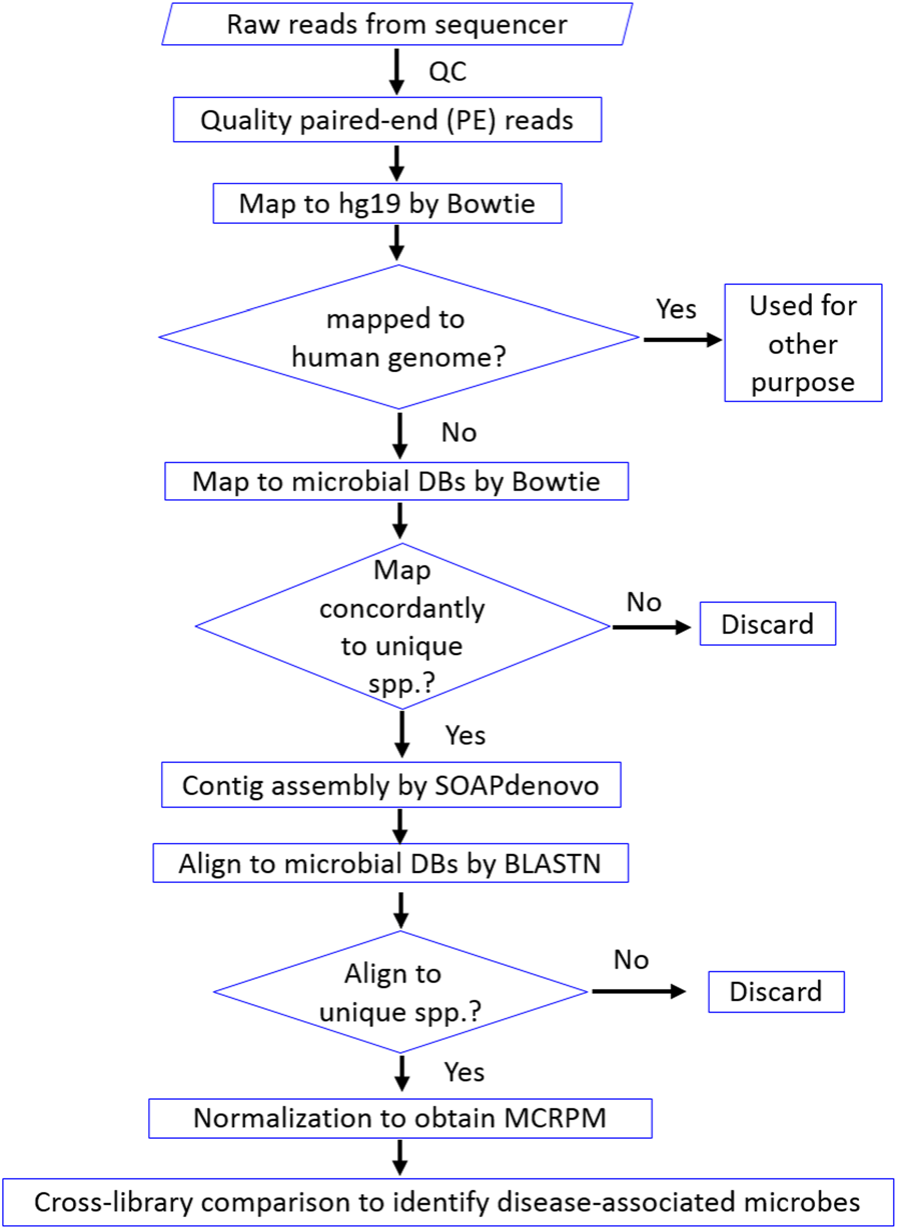
Workflow showing the stepwise procedure of sequence data processing leading to the identification of microbes in the body

Illumina sequencing adapter was trimmed by cutadapt. Both base quality and read quality were checked after contaminant reads were removed. PRINseq [27] was employed for base quality checking following the sequential steps: removal of low quality bases from both 5′- and 3′-ends, removal of reads having ≥ 3 ambiguous (N) bases, and removal of reads with read length < 30 bp. NGS QC Toolkit [28] was then used to select high quality reads each of which has base score ≥ 20 in ≥ 70% of the contained bases. A generalized workflow for data analysis is shown in Fig. 1.

### Mapping of PE reads against microbial databases

Quality PE reads were mapped to UCSC hg19 human genome assembly using bowtie2 under default parameters (maximum insert size defined as 800 bp). The unmapped PE reads were then used to map against bacterial, viral and fungal databases separately with bowtie2 under multiple hit output option (parameters: --end-to-end --no-mixed --no-discordant –k 2). The option “-k 2” defines the maximum number of hits as 2 (species) per PE read [29]. Since there exists a high degree of similarity among microbial sequences, we chose only PE reads that hit concordantly to a unique microbial species for contig assembly.

### Contig assembly and alignment for microbial species identification

Contigs were assembled with SOAPdenovo using Kmer 63 and then used to BLAST (BLASTN) against the same microbial databases with the following criteria: i) sequence identity ≥ 95%; ii) contig length ≥ 250 bp; and iii) contig aligned ratio (aligned length/contig length) = 100% (i.e., full range aligned). Strains belonging to the same genus/species were merged and number of contigs and total contig length were summed up accordingly.

### Normalization

Normalization is essential for intra- and inter-library comparison of microbial cfDNA level. It is calculated based on the number of reads, instead of the number of contigs associated with each microbial species. Notice that, the numbers of reads per contig may vary, and only the total read count, which correlates the total number of cfDNA fragments in plasma, is able to reflect the level of cfDNA fragments in plasma.

Thus, the numbers of sequence reads associated with all contigs that aligned to each species were summed up and normalized on “per-million quality reads” basis. This approach would not only reflect the relative levels of microbial cfDNA fragments in the original sample, but also allow us to weigh the relative significance among microbial cfDNA across all libraries.

### Statistical analysis

To compare the amount of microbial cfDNA in plasma between cancer patients and normal females, the average value of titer in MCRPM for each microbial species was calculated separately for normal females and diseased patients. Then, the averages for all bacteria were compared across these two groups using the non-parametric Mann-Whitney-Wilcoxon test.

## Abbreviations

cfDNA: Cell-free DNA
CTCs: Circulating tumor cells
EOBC: Early-onset breast cancer
MCRPM: Microbial cfDNA reads per million quality PE reads
NGS: Next-Generation Sequencing
PE: Paired-End
